# Increased thalamocortical connectivity to the medial prefrontal cortex with recovery of impaired consciousness in a stroke patient: A case report

**DOI:** 10.1097/MD.0000000000019937

**Published:** 2020-05-01

**Authors:** Sung Ho Jang, You Sung Seo, Sung Jun Lee

**Affiliations:** Department of Physical Medicine and Rehabilitation, College of Medicine, Yeungnam University, Daemyungdong, Namku, Taegu, Republic of Korea.

**Keywords:** ascending reticular activating system, consciousness, diffusion tensor tractography, intracerebral hemorrhage, stroke

## Abstract

**Rationale:**

We report a stroke patient who showed increased thalamocortical connectivity to the medial prefrontal cortex (mPFC) with recovery of impaired consciousness that was demonstrated on diffusion tensor tractography (DTT) of the ascending reticular activating system (ARAS).

**Patients concerns:**

A 48-year-old male patient underwent craniectomy and hematoma removal for spontaneous intracerebral hemorrhage in the right basal ganglia and thalamus. When he started rehabilitation at 5 weeks after onset he was in a vegetative state with a Coma Recovery Scale-Revised score of 6.

**Diagnoses:**

The patient was diagnosed spontaneous intracerebral hemorrhage in the right basal ganglia and thalamus.

**Interventions:**

He underwent comprehensive rehabilitation including neurotropic durgs, transcranial direct current stimulation, and repetitive transcranial magnetic stimulation of the left prefrontal lobe (Brodmann area 10).

**Outcomes:**

After 5 weeks of rehabilitation, the patient had recovered to a nearly normal conscious state with a Coma Recovery Scale-Revised score of 22. On 10-week DTT, thickening of the lower dorsal ARAS was observed on both sides compared with 5-week DTT. Decreased neural connectivity to the left PFC was observed on 5-week DTT whereas decreased neural connectivity to the left PFC was increased on 10-week DTT, especially the mPFC.

**Lessons:**

Increased thalamocortical connectivity to the mPFC was demonstrated in a stroke patient who showed concomitant recovery from a vegetative state to a nearly normal conscious state. The results suggest that the increased neural connectivity to the mPMC contributed to recovery of consciousness in this patient.

## 1 Introduction

Studies of relevant neural structures for the recovery of impaired consciousness are clinically important because such knowledge is mandatory to development of therapeutic strategies and prognosis prediction. The prefrontal cortex, which is mainly involved in cognitive processing, has been reported as a core neural structure involved in recovery of impaired consciousness.^[1–5]^ Among the components comprising the prefrontal cortex, the medial prefrontal cortex (mPFC) is a part of the default mode network of the brain that participates in various cognitive functions affecting human identification processes, altering attentional process, decision-making, and goal-directed behavior.^[2,6]^ The mPFC is also involved in consciousness, and recent studies have demonstrated that it is associated with consciousness recovery.^[2–4]^ However, it has not yet been clearly elucidated.

In this study, we report a stroke patient who showed increased thalamocortical connectivity to the mPFC with recovery of impaired consciousness that was demonstrated on diffusion tensor tractography (DTT) of the ascending reticular activating system (ARAS).

## 2 Case report

**Figure 1:**
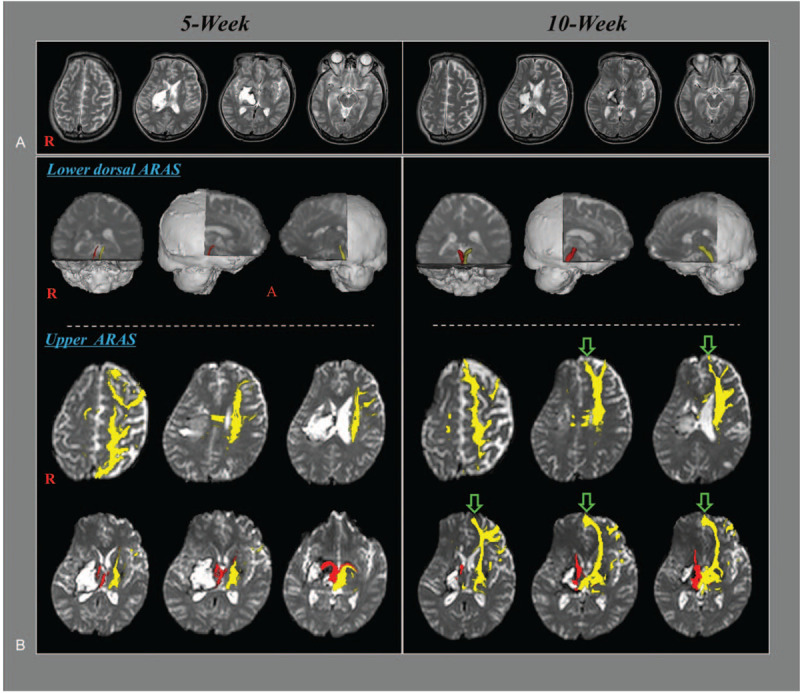
(A) Brain magnetic resonance images at 5 and 10 weeks after onset show leukomalactic lesions in the right basal ganglia and thalamus. (B) Results of diffusion tensor tractography (DTT) of the ascending reticular activating system (ARAS). On the 10-week DTT, thickening of the lower dorsal ARAS is observed on both sides when compared with the 5-week DTT. Decreased neural connectivity to the left prefrontal cortex is observed on the 5-week DTT whereas the decreased neural connectivity to the left prefrontal cortex is increased on the 10-week DTT, especially the medial prefrontal cortex (green arrows).

A 48-year-old male patient underwent craniectomy and hematoma removal for spontaneous intracerebral hemorrhage in the right basal ganglia and thalamus in the neurosurgery department of a university hospital. Approximately 5 weeks after onset, he was transferred to the rehabilitation department of the same university hospital. Brain magnetic resonance images at 5 and 10 weeks after onset showed leukomalactic lesions in the right basal ganglia and thalamus (Fig. 1-A). The patient was in a vegetative state (VS) with a Coma Recovery Scale-Revised (CRS-R) score of 6 (auditory function: 0, visual function: 3, motor function: 2, verbal function: 0, communication: 0, and arousal: 1).^[7]^ He underwent comprehensive rehabilitation, which included neurotropic drugs (modafinil, ropinirole, amantadine, levodopa, and baclofen) and physical and occupational therapies including tilt table standing. Transcranial direct current stimulation (tDCS) was also administered with a neuroConn DC-stimulator (neuroConn, Ilmenau, Germany).^[8]^ tDCS was applied using a battery-driven constant-current stimulator with saline-soaked surface sponge electrodes (7 cm × 5 cm). The anode was placed on the left mPFC, and the cathode was placed on the opposite supraorbital region. The stimulation intensity was 2 mA and the duration was 20 min/session with 1 session/day and 7 sessions/wk. Repetitive transcranial magnetic stimulation (rTMS) using a MagPro stimulator (Medtronic Functional Diagnostics, Skovlunde, Denmark) was applied to the left mPFC at a frequency of 10 Hz with an 80% motor threshold intensity and 160 pulses for 8 min/session with 1 session/d and 7 sessions/wk.^[9]^ After 5 weeks of rehabilitation, the patient had recovered to a nearly normal conscious state with a CRS-R score of 22 (auditory function: 4, visual function: 5, motor function: 5, verbal function: 3, communication: 2, and arousal: 3).^[7]^ The patient's wife provided signed, informed consent for publication, and approval for the study was obtained from the Institutional Review Board of Yeungnam University Hospital (approval no. YUMC-2019-06-032).

## 3 Diffusion tensor imaging

Acquisition of diffusion tensor imaging data was performed at 5 and 10 weeks after onset by using a 6-channel head coil on a 1.5 T Philips Gyroscan Intera (Philips, Best, Netherlands) with single-shot echo-planar imaging. Imaging parameters were as follows: acquisition matrix = 96 × 96; reconstructed matrix = 192 × 192; field of view = 240 mm × 240 mm; TR = 10,726 ms; TE = 76 ms; b = 1000 s/mm^2^; NEX = 1; and slice thickness of 2.5 mm with no gap (acquired isotropic voxel size 1.3 mm × 1.3 mm × 2.5 mm). For each of the 32 non-collinear diffusion sensitizing gradients, 67 contiguous slices were acquired parallel to the anterior commissure-posterior commissure line. Analysis of diffusion-weighted imaging data was performed using the tools within the Oxford Centre for Functional Magnetic Resonance Imaging of the Brain Software Library (www.fmrib.ox.ac.uk/fsl). Affine multi-scale 2-dimensional registration was used for correction of head motion effects and image distortion because of eddy currents. Fiber tracking was implemented by applying a probabilistic tractography method based on a multifiber model and was applied in the current study by utilizing tractography routines implemented in the functional magnetic resonance imaging of the brain Diffusion software (5000 streamline samples, 0.5 mm step lengths, curvature thresholds = 0.2). Two portions of the ARAS were reconstructed by selection of fibers passing through regions of interest (ROIs). For analysis of the lower dorsal ARAS, the seed ROI was placed on the pontine reticular formation, and the target ROI with the option of termination was placed on the thalamic intralaminar nucleus (ILN) at the level of the intercommissural plane between the anterior and posterior commissures.^[10]^ For reconstruction of the upper ARAS (the neural connectivity of the thalamic ILN to the cerebral cortex), the seed ROI was placed on the thalamic ILN.^[11]^ Out of 5000 samples generated from the seed voxel, contact results were applied at a minimum threshold for lower dorsal ARAS of 2 and neural connectivity of the upper ARAS of 10, which were streamlined through each voxel for our analysis.

On 10-week DTT, thickening of the lower dorsal ARAS was observed on both sides compared with those of 5-week DTT. Decreased neural connectivity to the left PFC was observed on 5-week DTT, whereas decreased neural connectivity to the left PFC was increased on 10-week DTT, especially the mPFC.

## 4 Discussion

In this study, serial DTTs were used to demonstrate changes in the lower dorsal ARAS between the pontine reticular formation and the thalamic ILN and in the upper ARAS between the thalamic ILN and the cerebral cortex in a stroke patient who showed concurrent recovery from a VS with CRS-R score of 6 at 5 weeks after onset to a nearly normal conscious state with CRS-R score of 22 at 10 weeks after onset during 5 weeks of rehabilitation. The main changes were observed in the upper ARAS between 5 and 10 weeks after onset and included increased thalamocortical connectivity to the left PFC (especially the mPFC). The decreased neural thalamocortical connectivity to the left PFC although the left side was unaffected appeared to be because the left thalamic injury was due to compression and the right was a result of thalamic hematoma. These results together with those of previous studies indicate that the increased thalamocortical connectivity to the left mPFC, which is an important area of the brain for consciousness, was mainly responsible for recovery from a VS to a nearly normal conscious state.^[2–4]^ Because the patient underwent rTMS and tDCS on the left mPFC every day for 5 weeks, we believe that tDCS and rTMS might have contribued to recovery of the thalamocortical connectivity to the left mPFC.

A few resting-state functional magnetic resonance images (fMRI)-based studies have demonstrated that the mPFC is related to consciousness in patients with acquired brain injury (ABI).^[2,12,13]^ In 2015, Wu et al found that the functional connectivity in the mPFC along with the precuneus, posterior cingulate cortex, and lateral parietal cortex was correlated with consciousness level and recovery outcome at 3 months after fMRI scanning in 99 ABI patients with varying degrees of consciousness loss.^[13]^ In 2017, Silva et al demonstrated functional connectivity between the mPFC and the posterior cingulate cortex could become a predictor for recovery of consciousness at 3 months after onset in 27 comatose patients with ABI.^[12]^ During the same year, Liu et al found that functional connectivity to the mPFC could serve as a mark to track the severity and outcome at 3 months after fMRI scanning in 17 patients with minimally conscious or VS.^[2]^ A few studies have reported increased thalamocortical connectivity to the mPFC with the concurrent recovery of impaired consciousness based on diffusion tensor imaging .^[3,4]^ In 2016, Jang et al reported a patient who showed recovery of impaired consciousness from a MCS to a normal state with recovery of the injured lower dorsal, ventral and upper ARAS (both mPFC) during a three-week period in the early stages of anoxic brain injury.^[3]^ Recently, Jang and Lee reported a stroke patient who showed recovery of consciousness from a minimally conscious state at 1 month after onset to a nearly normal conscious state at 7 months after onset with recovery of the injured lower dorsal, ventral, and upper ARAS (the mPFC).^[4]^

In conclusion, increased thalamocortical connectivity to the mPFC was demonstrated in a stroke patient who showed concomitant recovery from a VS to a nearly normal conscious state. These results suggest that increased neural connectivity to the mPMC contributed to the recovery of consciousness in this patient. We believe these observations may be important to the development of rehabilitative strategies for patients with impaired consciousness. However, some limitations of this study should also be considered. First, this study is limited because it is a single case report. Second, regions of fiber complexity and crossing can prevent full reflection of the underlying fiber architecture by DTT; therefore, DTT may underestimate the fiber tracts.^[14]^

## Author contributions

**Conceptualization:** Sung Ho Jang.

**Methodology:** Sung Jun Lee.

**Supervision:** Sung Ho Jang.

**Visualization:** You Sung Seo, Sung Jun Lee.

**Writing – original draft:** Sung Ho Jang.

**Writing – review & editing:** Sung Ho Jang, Sung Jun Lee.
